# The economics of decision-making in dementia care and related life-limiting illness: a narrative review

**DOI:** 10.1186/s13561-026-00803-2

**Published:** 2026-06-05

**Authors:** Ana Maksimovic, Simona Grassi, Clare Ellis-Smith, Peter May

**Affiliations:** 1 Cicely Saunders Institute of Palliative Care, Policy & Rehabilitation, Florence Nightingale Faculty of Nursing, Midwifery & Palliative Care, King’s College London, London, SE5 9PJ UK; 2 Department of Economics, King’s Business School, King’s College London, London, WC2B 4BG UK; 3 Centre for Health Policy & Management, School of Medicine, Trinity College Dublin, Dublin, D02 YD28 Ireland

**Keywords:** Dementia, Decision-making, Uncertainty, Asymmetric information, Principal-agent problem.

## Abstract

**Background:**

Dementia represents a profound healthcare and economic challenge for the 21st century. A significant proportion of associated costs originate in decision-making between people with dementia, family carers, and health and social care practitioners. Economics is a decision science that may inform such choices, but the research evidence has not been collated and synthesised. We review the decision-making literature to analyse how economic insights can be applied to decision-making in life-limiting illness and identify findings applicable to dementia.

**Methods:**

Our literature search was comprehensive and included EconLit, Medline, Embase, and PsycInfo, as well as a targeted search of relevant economic literature.

**Results:**

We identified 9,854 studies, of which 62 were included. We found six key economic concepts: (a) principal-agent problem, (b) uncertainty, (c) asymmetric information, (d) hyperbolic discounting, (e) the empathy gap, and (f) non-linear value of time. These findings are helpful in understanding established patterns in the evidence base, including high end-of-life costs and variable efficacy of advance care planning.

**Discussion:**

These findings are used to consider the modifiability of formal, social, and household costs, and identify opportunities for future research. Dynamic communication between all decision-makers (patient, family, clinicians) is essential to overcome the identified barriers and drive improvements in dementia care. Further, we note that by integrating economic insights with lived experience, health sciences researchers get a better understanding of how decisions are made.

**Conclusion:**

Examining the dynamics that shape individual-level decision-making promotes a better understanding of the drivers of costs in life-limiting illness. Economics should make wider use of this methodological approach to examine decision-making in dementia care.

**Supplementary Information:**

The online version contains supplementary material available at 10.1186/s13561-026-00803-2.

## Background

Dementia is widely recognised as a priority economic issue [1, 2]. Global societal costs have been estimated at $1313.4 billion USD, spread across formal health care systems (16%), social care (34%), and opportunity costs associated with informal care and loss of labour (50%) [3]. Beyond the economic costs, people living and dying with dementia and their family carers often experience a decline in quality of life – a deficit that also carries an implicit cost [4].

Dementia costs are of increasing concern due to rising prevalence. The number of people with dementia is estimated to rise from 57.4 million in 2019 to 152.8 million by 2050 [5]. As the demand for dementia care grows, costs are rising more quickly still due to increasing real input costs and growing patient complexity [6, 7]. Therefore, there is a pressing need for economics research to tackle the growing costs of dementia care in high-income countries and low- and middle-income countries alike [3, 8].

As a proportion of costs fall upon formal systems, and an even larger amount on family carers, whose availability mediates the demand for formal care, this is naturally a significant area of interest for health systems [9, 10]. Cost-effectiveness analysis (CEA) is a core tool of health economics for evaluating care and is being widely applied in the search for cost-effective medications [11]. However, medications only account for a small proportion of the societal costs of dementia. Instead, societal costs more heavily reflect the aggregated outcomes of the countless decisions made by people with dementia, health and social care practitioners, and informal carers such as family and friends [12]. Economics as a decision science holds the potential to provide insight into how information and preferences shape care choices and drive the costs of dementia.

The limitations of traditional economic assumptions in a general medical care context have long been recognised. In his seminal paper, Arrow (1963) [13] enumerated these limitations as including the unpredictable nature of individual-level demand, unacceptability of supply-side profit-maximising behaviour, product uncertainty, supply-side barriers to entry and extensive price discrimination by income. The relevance of these observations persist, but additional dynamics are observable in the context of ageing populations with high prevalence of serious life-limiting illness. Some dynamics may be an incremental extension of concepts previously identified; for example, information asymmetry has specific relevance and meaning in the context of cognitive decline. Other aspects may be altogether more complex; for example, the prominent role of family carers potentially introduces additional knowledge, uncertainty and preferences to decision-making, as well as having wider economic implications for workforce participation and welfare.

The purpose of this study is to collate the decision-making literature in serious life-limiting illness, synthesise the identified economic concepts that underpin decision-making in this clinical context, and examine how these insights apply to decision-making in dementia. In doing so, we aimed to highlight the potential of economic insights beyond CEA to contribute to understanding of the drivers of high societal costs. We identify opportunities for future research by discussing the modifiability of formal, social and informal care costs.

## Methods

### Study design

We chose a narrative review design to enable interpretation and synthesis of a wide and heterogenous body of literature across disciplines [14]. This approach allowed us to describe what is known while critically examining and integrating evidence to generate new insights, reflecting the unique perspective of the review team [15]. We systematically searched the decision-making literature broadly, applying an economic ‘mode of thinking’ to interpreting the results [16]. Coast and De Allegri (2018) [16] have defined an economic mode of thinking as applying core economic concepts such as information and preferences to various settings where these concepts are relevant. We also utilised purposive identification of economics literature relevant to themes from the systematic search. Patient and public involvement (PPI) members with dementia, or experience of caring for someone with dementia, were consulted during workshops on the progress of the study and invited to discuss findings.

### Search strategy

We searched health and social care (Medline, Embase, PsycINFO via Ovid) and economics (EconLit via ProQuest) electronic databases for articles published by 31/12/2024 and then conducted an updated search until 31/12/2025. Databases were searched from inception to avoid excluding early contributions relevant to decision-making in dementia. For different topic databases, we used distinct but complementary search strategies to identify articles. The search strategy was developed with the guidance of an academic librarian experienced in systematic searches to ensure appropriate database selection and search terms. Full details are provided in the supplementary materials.

Studies were classified as economics or health and social care studies based on if they were indexed in SCOPUS under ‘Economics, Econometrics and Finance’ or listed in the Academic Journal Guide 2024 under the field ‘ECON’. Grey literature sources were assessed individually to ensure that studies explicitly identified themselves as economics research.

### Eligibility criteria

We aimed to identify economic insights relevant to decision-making from diagnosis to end of life for adults with any type of dementia, including those with multimorbidity. This meant that we were not interested in the prevention or screening of conditions, but in how people and their families manage the condition from diagnosis [17]. Given that understanding of and research into the decision-making processes for people with dementia is new relative to other progressive diseases, we anticipated that a single-disease focus would yield a small literature. Moreover, we hypothesised that the larger body of work in serious life-limiting illness, defined as incurable conditions that often involve palliative care, such as cancer, advanced organ disease, and HIV, would contribute dementia-relevant insights [18]. Therefore, we widened our search to life-limiting illness, and in analysis we refined the identification of themes and interpretation of findings to prioritise qualities most relevant to dementia care. Interpretation was informed by reflections from PPI members on the relevance of these findings to lived experience.

We developed our eligibility criteria to identify studies focusing on dementia, or conditions with similar characteristics to dementia, which is defined as a progressive, incurable condition primarily affecting adults and associated with cognitive impairment [2]. Summarised inclusion criteria were as follows: (a) published by 31/12/2025, (b) life-limiting conditions (incurable and progressive) or cognitive impairment, (c) participants are at least 18 years old, (d) ongoing management, treatment and care interventions with palliative or supportive intent. We excluded conference abstracts and study protocols. Beyond this, no further restrictions on study design were imposed in order to capture the methodological range of research across disciplines. No restrictions on language were imposed.

### Study selection

Studies identified via the systematic search were imported into Covidence, the standard online platform for screening evidence. Duplicates were initially removed automatically by Covidence, followed by manually screening to identify any remaining duplicates. Titles/abstracts were screened by AM to exclude irrelevant studies. Eligible studies were subsequently retained for full-text screening. This was conducted by one researcher (AM) independently and repeated independently for 20% of studies by another researcher (PM). Following completion of the systematic search, additional studies were identified by both AM and PM to provide further insight into themes that emerged from the systematic search. Any discrepancies were discussed between AM and PM until agreement was reached.

### Data extraction and analysis

Data were extracted from selected studies and a spreadsheet was used to document themes and results. Due to the variation in methods between the health and social, and economics, literature, we employed thematic synthesis to bridge together the disciplines [19]. Themes were developed inductively following completion of full-text screening, based on recurring concepts identified within the included economics studies. These themes were used to interpret the included health and social care literature, even when not expressed in identical terms. These processes were conducted by AM and repeated for 20% of studies by PM. As with the full-text screening, discrepancies were resolved between AM and PM. PPI members informed the interpretation of the results by highlighting findings most relevant to dementia.

## Results

### Study selection

**Fig. 1:**
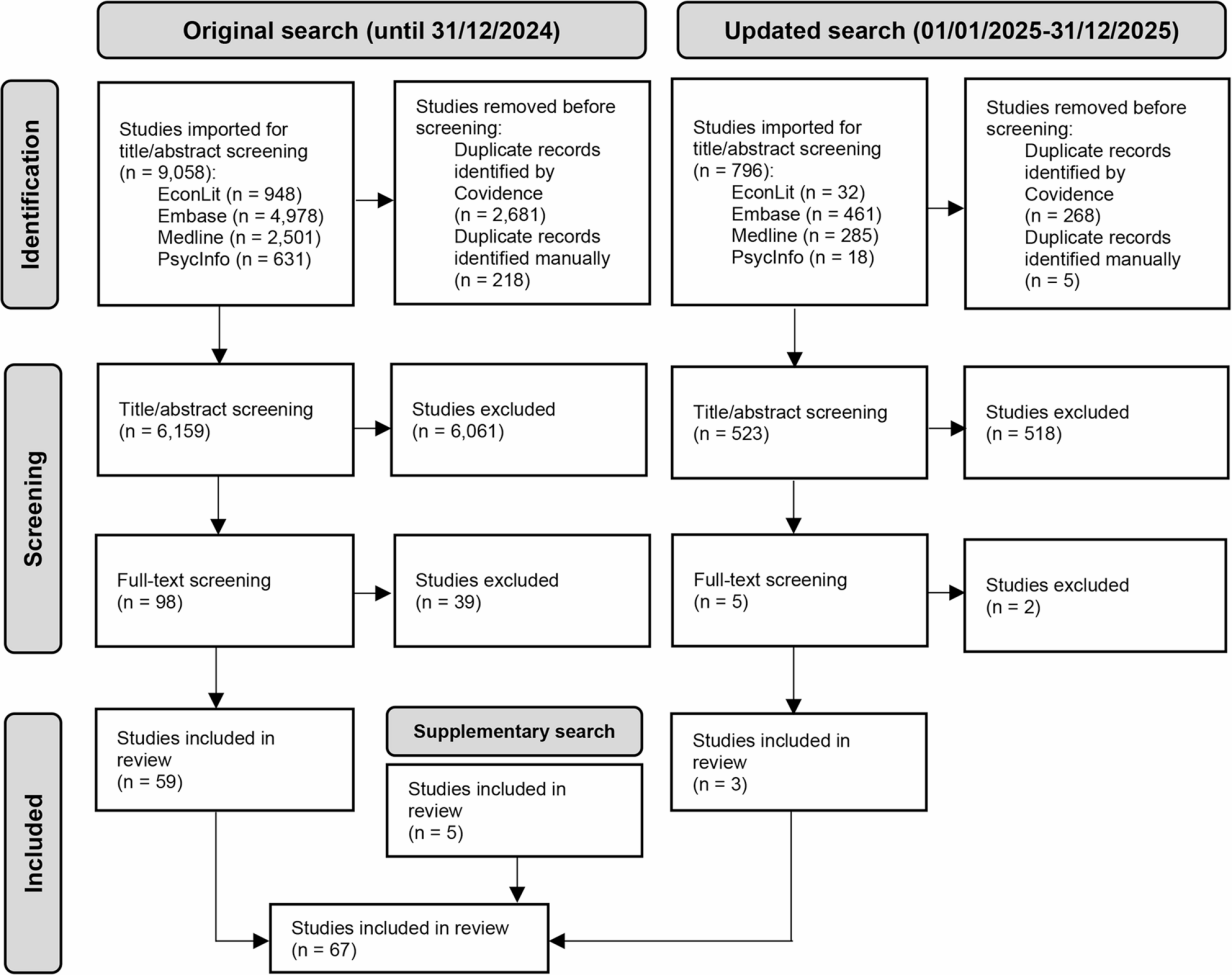
Flow chart for systematic search

In the original search, 9,058 articles were identified from the database search, which was reduced to 6,159 after removing duplicates. 98 studies were included in full-text screening, and finally 59 studies were included in the review. In the updated search, an additional 796 articles were identified, resulting in a further three studies being included. Overall, 62 studies were identified in the systematic search. A further five studies were identified in the supplementary search (Fig. 1).

### Summary of included studies

Of the 62 studies included, 37 (60%) were from health and social care journals and 25 (40%) were from economics journals (Table 1). 26 (42%) studies were based in the USA and 11 (18%) in the UK with the remaining 25 studies coming from a total of 14 other countries. The conditions explored were mainly dementia (37%), cancer (16%), cognitive impairment (13%), end-of-life conditions (10%), life-limiting illness (8%), chronic kidney disorder (5%), and both dementia and cancer (3%).

**Table 1:** Summary of decision-making studies in serious life-limiting illness

Author/Year (country)	Illness	Study Design and Aim	Main Findings (Theme(s))	PPI
Economics Literature				
Allard et al., 2011 (Canada) [20]	Life-limiting illness	Comparative study comparing incentive properties of GP payment mechanisms	Fee for service and fundholding associated with fewer specialty care referrals than capitation *(principal-agent problem)*	NR
Amsterlaw et al., 2006 (USA) [21]	Cancer	Experimental study comparing preferences for uncomplicated (20% mortality) vs. complicated (16% mortality and 4% complication risk) surgery	Many participants chose uncomplicated surgery despite preferring survival over complications *(asymmetric information)*	NR
Angrisani and Lee, 2019 (USA) [22]	Cognitive impairment	Secondary data analysis examining association between cognition and financial outcomes in older couples	Cognitive decline associated with greater wealth loss when affected spouse was financial decision-maker and transfer of decision-making to the non-impaired spouse *(principal-agent problem)*	NR
Becker et al., 2007 (USA) [23]	Terminal illness	Working paper rationalising futile spending on end-of-life care	High spending is rational because the value of a life year increases at end of life due to hope *(non-linear value of time)*	NR
Buckell et al., 2022 (USA, Canada, South Africa) [24]	HIV	Empirical study comparing heterogenous and single decision rule models	Utility maximisation models were overall preferred, but individuals apply varying decision rules *(uncertainty)*	NR
Byrne et al., 2009 (USA) [25]	People aged 70 and over	Game-theoretic modelling and secondary data analysis of family decision-making regarding formal and informal care	Family caregiver’s utility depends on their own and their relative’s provision of care *(uncertainty)*	NR
Chandra et al., 2021 (USA) [26]	Dementia	Theoretical study identifying future areas of economics research	Future economics research should explore decision-making with cognitive impairment, highlight research and development incentives, and identify ways to improve long-term care *(principal-agent problem; uncertainty)*	NR
Felder, 2020 (Switzerland) [27]	End-of-life	Theoretical study analysing effects of health status, wealth and mortality risk on the propensity to treat under uncertainty	Treatment propensity increases with mortality risk but may decrease with illness severity *(uncertainty; non-linear value of time)*	NR
Hsu and Willis, 2013 (USA) [28]	Dementia	Secondary data analysis examining how information on cognitive decline influences selection of the financial decision-maker	Declining cognition in financial decision-maker leads to the transfer of the role to the non-cognitively impaired spouse, though often delayed. A diagnosis accelerates the transition *(principal-agent problem)*	NR
Huffman et al., 2019 (USA) [29]	Cognitive impairment	Empirical study examining association between time discounting and economic behaviours in older adults	Cognitive decline associated with impatience, which was associated with reduced wealth, fewer health investments, and less end-of-life planning *(hyperbolic discounting)*	NR
Keane et al., 2016 (Australia) [30]	Cognitive impairment	Review of complex decision-making affecting the elderly	Elderly consumers can struggle to make optimal choices due to limited understanding of options or cognitive capacity to evaluate payoffs *(uncertainty)*	NR
Kvaerner, 2023 (Norway and the Netherlands) [31]	Life-limiting illness	Empirical study examining inter vivo transfer and bequest decisions following negative news regarding life expectancy	Negative news regarding life expectancy increases probability and size of wealth transferred to the next generation *(asymmetric information)*	NR
Lomas and Lavis, 1996 (Canada) [32]	Gray zone illness	Working paper examining the gray zone of medical care and guidelines	A quarter to a third of care falls in a gray zone, where evidence is limited or preference-dependant *(asymmetric information)*	NR
Mazzonna and Peracchi, 2024 (USA) [33]	Cognitive impairment	Secondary data analysis examining awareness of cognitive decline in older people and its financial consequences	Older people often underestimate their cognitive decline and tend to suffer wealth losses *(asymmetric information)*	NR
Mazzonna and Peracchi, 2012 (Europe) [34]	Cognitive impairment	Secondary data analysis examining association between ageing, cognitive abilities, and retirement	Retirement may increase cognitive decline by reducing incentives to invest in cognitive stimulation *(principal-agent problem)*	NR
Nicholas et al., 2024 (USA) [35]	Dementia	Experimental study examining surrogate end-of-life decisions and the impact of dementia and advance planning	Respondents less likely to choose life-sustaining treatments (LET) for people with dementia and ignore preferences for LET. When patient preference unclear, respondents more likely follow their own preferences *(asymmetric information; empathy gap)*	NR
Philipson et al., 2010 (USA) [36]	Terminal illness	Working paper rationalising futile spending on end-of-life care	High spending is rational because the value of a life year increases at end of life due to hope (*non-linear value of time)*	NR
Pilny and Stroka, 2016 (Germany) [37]	Long-term care recipients	Empirical study examining determinants of received long-term care	Decision for long-term in-patient care is associated with supply of nursing home beds *(uncertainty)*	NR
Roan Gresenz et al., 2025 (USA) [38]	Dementia	Longitudinal study examining spillover effects of cognitive decline in one member of coupled household on financial outcomes of their partners	Dementia affects the financial outcomes of both the person living with dementia and their partner *(principal-agent problem)*	NR
Richards, 2013 (USA) [39]	End-of-life	Theoretical study of rationality in modern economic and Ancient concepts	Eudaimonic concept of the good could lead to a more efficient health care system *(uncertainty)*	NR
Round, 2012 (UK) [40]	End-of-life	Theoretical study addressing criticisms of QALYs in end-of-life and palliative care	QALYs remain the best available outcome measure *(non-linear value of time)*	NR
Sheng, 2022 (Denmark) [41]	Chronic conditions	PhD thesis examining effect of patient-physician socio-economic status (SES) concordance on difference in mortality between high- and low-SES patients	SES concordance decreases the difference in mortality between high- and low-SES patients *(asymmetric information)*	NR
Woosley et al., 2017 (USA) [42]	End-of-life	Secondary data analysis examining impact of adult child’s environment and perceptions, and parent’s end-of-life choices, on end-of-life planning	Adult children’s end-of-life planning most influenced by household net worth, avoidance of thoughts of death, and parent’s completion of living will *(hyperbolic discounting)*	NR
Xie et al., 2025 (China) [43]	Cancer	Secondary data analysis examining the effect of a cancer diagnosis on the spouse’s healthcare behaviours	Following their partner’s diagnosis, the spouse’s healthcare utilisation, information on the disease, and personal health awareness increase *(asymmetric information)*	NR
Ye and Yi, 2023 (Southeast Asia) [44]	Emergency department patients	Secondary data analysis examining impact of patient-physician race concordance on physician decision-making and patient health outcomes	Patient-physician race concordance led to longer consultations, fewer inpatient admissions and diagnostic testing, and fewer emergency department revisits *(asymmetric information)*	NR
Health and Social Care Literature				
Anantapong et al., 2023 (UK (England)) [45]	Dementia	Qualitative study examining hospital patients’ eating and drinking decisions to modify a decision-making model	Decision-making requires shared input and inclusion of everybody’s values, but hospital staff often act without consulting family carers *(principal-agent problem; uncertainty; asymmetric information)*	Yes
Baum et al., 2021 (USA) [46]	Dementia or cognitive impairment	Secondary data analysis comparing end-of-life care when children vs. spouses are involved in decision-making	Patients less likely to receive life-extending treatments when the spouse was involved *(asymmetric information)*	NR
Beckwith et al., 2023 (UK) [47]	Kidney failure	Scoping review exploring life expectancy expectations	Patients predicted higher life expectancy than professionals, and those with lower perceived life expectancy preferred comfort-care *(hyperbolic discounting)*	NR
Bluhm et al., 2016 (USA) [48]	Cancer	Qualitative study exploring the self-reported factors influencing oncologist’ decision to prescribe chemotherapy	Clinical guidelines shape decisions when treatment options are clear, while emotions are salient when clinical factors are ambiguous. Oncologists prescribe late chemotherapy to manage the strain of communication, responsibility, prognostic uncertainty and limited healing ability, and to maintain hope *(uncertainty; hyperbolic discounting)*	NR
Bolt et al., 2016 (The Netherlands) [49]	End-of-life	Qualitative study exploring patients’ and relatives’ perception of appropriate and inappropriate end-of-life care	Appropriate care supports patient’s wishes, hope, and communication. Dementia care prioritises support and location. Inappropriate care involves overtreatment and poor communication *(non-linear value of time)*	NR
Boogaard et al., 2017 (Israel) [50]	Dementia	Quantitative study assessing family caregiver trust in nursing home health professionals and associated outcomes	Half to one-third of family caregivers reported moderate-to-low trust. Trust associated with higher family satisfaction and positive evaluations of physician-family communication *(asymmetric information)*	NR
Bosco et al., 2019 (UK) [51]	Dementia	Systematic review and meta-ethnography exploring agency of people living with dementia	Agency varies between autonomous, shared, and pseudo decision-making *(principal-agent problem; uncertainty)*	NR
Boucher et al., 2019 (Canada) [52]	Cognitive impairment	Secondary data analysis examining factors associated with informal caregiver burden among those who made housing decisions	Caregiver burden associated with female caregivers, decision regret/conflict, preference for loved one to move into a caregiver’s or nursing home, made decision recently, and perceived joint decision-making *(asymmetric information)*	NR
Brazil et al., 2018 (UK (Northern Ireland)) [53]	Dementia	Randomised controlled trial evaluating effectiveness of advance care planning with family caregivers	Advance care planning reduced family caregiver uncertainty and improved perceptions of quality of care *(asymmetric information; non-linear value of time)*	NR
Brown et al., 2016 (UK) [54]	Advanced kidney disease	Commentary outlining communication strategies to improve cross cultural competency	Shared decision-making promotes culturally competent clinician communication *(asymmetric information)*	NR
Caba et al., 2021 (USA) [55]	Dementia and cancer	Mixed studies review assessing impact of dementia on cancer decision-making, treatment, and mortality	Dementia increased chemotherapy, radiation, and surgery. Patients with cancer and dementia had higher mortality than those with only cancer *(asymmetric information)*	NR
Chochinov, 2011 (Canada) [56]	End-of-life	Commentary rationalising high end-of-life spending	End-of-life spending is rational as value of time increases at end of life *(uncertainty; non-linear value of time)*	NR
Cresp et al., 2020 (Australia) [57]	Dementia	Systematic review and qualitative meta-synthesis exploring surrogate decision-makers experience	Surrogate decision-makers experience mistrust in healthcare professionals, guilt, and uncertainty in advance care plans, limited knowledge, communication issues, and end-of-life uncertainties *(uncertainty; hyperbolic discounting)*	NR
Davies et al., 2021 (UK (England)) [58]	Dementia	Qualitative study modifying model of decision-making	Modified the Interprofessional Shared Decision Making model to include preferences, advance care planning, wellbeing, support from others, and clarity of role *(principal-agent problem; uncertainty)*	Yes
De Kort et al., 2010 (The Netherlands) [59]	Cancer	Qualitative study investigating the process of patient-oncologist decision-making	Decision-making is an erratic process, continually revised based on patient response, experience, and changes in disease *(principal-agent problem; uncertainty)*	NR
Dening et al., 2016 (UK) [60]	Dementia	Quantitative study examining whether family caregivers’ choices align with patient preferences and associated factors	Choices align for current health states but diverge more for future hypothetical health states, where carers favour more intervention *(empathy gap)*	NR
Epstein, 2021 (USA) [61]	Life-limiting illness	Theoretical study exploring epistemic uncertainty for clinicians and patients	Discomfort with uncertainty leads patients and clinicians to supress death-related thoughts *(uncertainty)*	NR
Fowler et al., 2013 (USA) [62]	Dementia and mild cognitive impairment	Secondary data analysis measuring perceived caregiver involvement in decision-making and anticipatory grief	64% of caregivers reported involvement in medical decision-making, while anticipatory grief impaired problem solving *(hyperbolic discounting)*	NR
Hossain et al., 2019 (UK (England)) [63]	Dementia	Qualitative study exploring perspectives of Bangladeshi family caregivers	Most caregivers lacked knowledge and awareness of dementia symptoms *(asymmetric information)*	NR
Hynes et al., 2022 (UK) [64]	Dementia and cancer	Qualitative study exploring caregiver experience in cancer decision-making for people with dementia	Services struggle to support people with both dementia and cancer, while caregivers report lack of support and poor understanding *(asymmetric information)*	Yes
Jawed et al., 2025 (USA) [65]	Chronic kidney disease	Survey-based study comparing decision-making skills and treatment attitudes of nephrologists and patients	Most nephrologists are comfortable with paternalistic and shared decision-making, though report difficulties in discussing palliative care *(hyperbolic discounting)*	NR
Kim et al., 2024 (South Korea) [66]	Dementia	Integrative review exploring uncertainty in surrogate decision-makers	Surrogates have negative response to uncertainty, but process it with relatives and health and social care practitioners *(uncertainty)*	NR
Laidsaar-Powell et al., 2017 (Australia) [67]	Cancer	Theoretical study proposing a framework of triadic decision-making that includes family caregivers	Caregiver influence varies within and across triads, and decisions can involve wider networks *(principal-agent problem)*	Yes
Malhotra et al., 2015 (Singapore) [68]	Cancer	Discrete choice experiment comparing patients’ and caregivers’ willingness to pay (WTP) for one more year of patient’s life vs. other end-of-life improvements	Patient’s WTP for life extension was similar to avoiding pain, dying at home, not burdening family and friends, or receiving quality care, while caregivers showed higher WTP for all *(asymmetric information)*	NR
Miller et al., 2016 (USA) [69]	Dementia	Review of patient and family caregiver decision-making processes	Not all people with dementia are excluded from decision-making, but the extent varies *(principal-agent problem)*	NR
Monnet et al., 2024 (Europe) [70]	Dementia	Qualitative study revising advance care planning definition using people with dementia, their relatives and friends, and staff members	Definition adds decreasing decisional capacity, family and/or trust-based relationships, and social aspects of care. Maintains emphasis on continuity, optional participation, communication, and documentation *(hyperbolic discounting)*	Yes
Mukadam et al., 2011 (UK) [71]	Dementia	Qualitative study examining attitudes on help-seeking in minority ethnic and indigenous groups	Minority ethnic groups, unlike indigenous groups, delayed help-seeking until they could no longer cope or others commented, viewing their role as a duty *(asymmetric information)*	NR
Nicholas et al., 2023 (USA) [72]	Dementia	Secondary data analysis examining whether surrogates and advance directives (AD) influence decisions	Surrogate involvement reduced burdensome treatments, transfers, and in-hospital death, regardless of number of decision-makers or AD *(asymmetric information)*	NR
Nierop-van Baalen et al., 2024 (The Netherlands) [73]	Cancer	Systematic review identifying factors associated with hope	Hope positively associated with quality of life, social support, and spiritual well-being, and negatively with symptom burden, distress, and depression *(non-linear value of time)*	NR
Owsley et al., 2022 (USA) [74]	Dementia	Secondary data analysis comparing treatment preferences between adults with and without cognitive impairment	Cognitive impairment associated with greater uncertainty about treatment preferences and stronger wish for aggressive care among those who stated a preference *(uncertainty)*	NR
Pitkala et al., 2021 (Finland) [75]	Dementia	Secondary data analysis comparing dementia costs of living alone or with a caregiver to estimate value of informal caregiving from a societal perspective	Participants living alone incurred a higher cost than the overall average, while costs for those living with a spouse were significantly lower than the average *(principal-agent problem)*	NR
Reyna et al., 2024 (USA) [76]	Cognitive biases	Theoretical study applying fuzzy-trace theory to medical decision-making	People rely more on gists of information than on complete information on outcomes and probabilities in decision-making *(asymmetric information)*	NR
Roach et al., 2022 (USA) [77]	Dementia	Qualitative study examining how nursing home staff and proxies describe guilt as an influence on end-of-life decision-making	Nursing home staff noted that proxies feeling guilty about relationship or lack of time with loved one leads to more aggressive care. No proxies reported guilt, instead referencing obligation and commitment *(hyperbolic discounting)*	NR
Shin et al., 2018 (South Korea) [78]	Cancer	Quantitative study comparing patients’, family caregivers’ and physicians’ understanding of disease stage, treatment goal, and chance of cure	Agreement rates between patient-physician and caregiver-physician were 63% and 65.9% for disease stage, 69% and 70% for treatment goal, and 41% and 45% for chance of cure *(asymmetric information)*	NR
Smebye et al., 2021 (Norway) [79]	Dementia	Qualitative study exploring participation of people with dementia, family carers and professional caregivers in decision making	Shared decision-making was most common. Involvement of people with dementia varied, often limited by reduced mental capacity, lack of choices, and not being given the opportunity *(principal-agent problem)*	NR
Van de Water et al., 2024 (The Netherlands) [80]	Cancer	Secondary data analysis exploring association between shared decision making and patient adverse outcomes	Shared decision-making increased tension, hopelessness and decisional uncertainty one week after consultation, but had no impact on long-term outcomes *(hyperbolic discounting)*	NR
Von Grueningen et al., 2005 (USA) [81]	Cancer	Review of the clinical meaning of futility	Futile treatments have no physiologic, quantitative or qualitative meaningful purpose *(non-linear value of time)*	NR

Note: NR indicates that PPI was not reported

Note: main findings refer to the primary results reported by the study authors. These may not represent the specific aspects of each study that motivated inclusion in this review or the economic concepts applied

Of the 37 studies from the health and social care literature, 32% were based in the USA and 27% in the UK. Conditions were categorised into dementia (51%), cancer (22%), end-of-life conditions (5%), both dementia and cancer (5%), cognitive impairment (8%), chronic kidney disorder (8%), and life-limiting illness (3%). Five studies included PPI (14%).

Of the 25 studies from the economics literature, 56% of studies were based in the USA, and 4% in the UK. Conditions were categorised into cognitive impairment (20%), dementia (16%), life-limiting illness (16%), end-of-life conditions (16%), and cancer (8%). No studies included PPI.

### Main results

We identified six key economic concepts in the literature relevant to dementia: (1) principal-agent problem (14 studies, or 23%), (2) uncertainty (17, or 27%), (3) asymmetric information (22, or 35%), (4) hyperbolic discounting (10, or 16%), (5) the empathy gap (2, or 3%), and (6) non-linear value of time (9, or 15%). Each concept is now discussed in turn.

#### Principal-agent problem

The principal-agent problem describes the difficulties that arise when a principal (patient) relies on an agent (surrogate decision-maker) for decision-making support [26, 58, 79]. Differences in preferences, incentives, and values can result in choices misaligned with the principal’s preferences.

This concept may be particularly relevant in dementia, where cognitive decline impairs the ability to process information and increases reliance on others for decision-making support [34]. For example, people with dementia are more vulnerable to financial fraud and often transfer financial decision-making responsibilities to a carer [22, 26, 28]. Further, cognitive decline is shown to affect the financial outcomes of both the individual diagnosed and their partner [38]. As dementia progresses, people increasingly rely on health and social care practitioners and family carers to advise or make decisions on their behalf, resulting in greater carer burden and increased total societal costs of dementia [58, 75, 79].

In the economics literature, the principal-agent problem has mainly been applied to patient-physician dyads. However, in the health and social care literature, these decision-making dynamics have been modelled in various ways, with differing distributions of responsibilities.

Paternalistic, or pseudo, models depict health and social care practitioners making decisions on behalf of patients [20, 45, 51, 59]. In contrast, counselling, or autonomous, models describe the patient independently making decisions [59]. Shared decision-making, the model recommended by the NHS, sits in the middle, with patients, family carers, and health and social care practitioners collaboratively determining care plans [45, 51, 67, 69].

#### Uncertainty

Neoclassical economists have typically relied on expected utility theory to model decision-making under uncertainty, depicting individuals as rational, fully informed agents who make choices that maximise their utility given well-defined preferences and probabilities [24, 25, 27, 30, 37, 39]. This framework has long been recognised as having limitations in capturing the complexities of healthcare decisions since the likes of Arrow (1963) [13]. Dementia presents additional challenges to these assumptions as cognitive decline impairs individuals’ ability to process information, form stable preferences, and make consistent choices under uncertainty [51].

It is often uncertain how dementia will progress, how long the patient will live, or when the end-of-life phase begins [58]. The causes of dementia are not well understood, and dementia is underdiagnosed [26]. Even with a diagnosis, dementia progression introduces increasing uncertainty, as patients face more complex care decisions at the same time as their ability to process them declines [59].

People struggle to cope with uncertainty. In dementia, where patients may lose the ability to communicate their preferences, carers often struggle to make choices that align with those preferences [45, 61, 74]. When combined with limited health literacy, this can negatively impact carer quality of life. This is due to family carers experiencing an elevated sense of responsibility and decisional regret [56–58, 61, 66].

Health and social care practitioners respond particularly poorly to uncertainty. Medical schools praise aspiring healthcare professionals for having the right answer [61]. As many clinicians enter the profession with the desire to cure people, it can be uncomfortable to treat life-limiting illness [82]. This discomfort can lead clinicians to overprescribe aggressive treatments at end of life and avoid uncomfortable conversations about dying, thereby exacerbating information asymmetry [48, 61].

#### Asymmetric information

Healthcare decisions are characterised by asymmetric information, as clinicians typically possess greater knowledge about diagnoses, treatment options, and expected outcomes than patients and carers [13]. Beyond this informational imbalance, differences in individual values and preferences further complicate decision-making [21]. As Lomas and Lavis (1996) [32] note, many care choices fall within a ‘gray zone’ between clearly effective and ineffective options, where choices depend less on objective evidence and more on how preferences are reconciled.

In dementia care, the principal-agent problem arises because of asymmetry of knowledge and preferences between the patient (principal) who relies on health and social care practitioners, and family carers (agents) in decision-making. Particularly with the additional constraint of cognitive impairment, when the agent’s preferences diverge from those of the principal, decisions may reflect the agent’s values. Therefore, information asymmetry may inhibit preference concordant care, i.e. care that aligns with the person living and dying with dementia’s preferences.

Multimorbidity can further exacerbate information asymmetry. Caba et al., (2021) [55] found that patients with both dementia and cancer have more complex clinical guidelines than those with cancer alone. This may suggest why people with dementia are likely to receive a cancer diagnosis at an advanced stage, which in turn limits treatment options, worsening outcomes. Hynes et al., (2022) [64] observed such patients are often treated primarily as cancer patients. Owing to the limited guidance on managing this subgroup, they are less likely to be treated holistically.

Cultural background also shapes information asymmetry. In some contexts, such as Hindu communities and South Korea, family members are informed of a terminal diagnosis first and often withhold this information from the patient [54, 71, 78, 83]. In Spain and Italy, the patient often needs to explicitly request to be informed of a terminal diagnosis, while the Anglo-American norm of patient autonomy promotes a culture of truth-telling [54].

Cultural background can also aid the management of information asymmetry, as concordance in socio-economic background and race between patients and physicians has been shown to promote communication and trust [41, 44]. This points to the importance of trust as an enhancer of information exchange [50, 63].

Providing more information can be beneficial. For example, unawareness of one’s own cognitive decline is associated with household wealth losses [33]. Receiving negative news around life expectancy increases the probability and size of transferring wealth to the next generation, allowing individuals to prepare [31]. Further, diagnosis of a life-limiting illness can have positive spillover effects on loved ones. For instance, individuals whose partner receives a cancer diagnosis are found to seek more information about their own health status [43].

Advance care planning can be interpreted as an intervention for managing information asymmetry. It refers to discussions around goals of care and end-of-life planning, often documented in an advance directive, and is related to shared decision-making [45, 53]. By sharing patient preferences, this should improve concordance amongst decision-makers [46].

However, providing more information can have adverse effects. Some studies show that when health and social care practitioners share clinical information with informal carers in an effort to promote shared decision-making, this increases burden [52, 64, 76]. Rather than feeling empowered, informal carers experience heightened uncertainty in making the right choice. Regarding advance care planning, Brazil et al., (2018) [53] found that it does not reduce informal carer burden, aggressive end-of-life care, or the number of hospitalisations or deaths in hospital. Studies show that advance directives are often ignored, or overridden with family carer preferences, suggesting that such documents alone cannot overpower the principal-agent problem [35, 72].

Therefore, providing more information does not ensure individuals will make decisions aligned with their own stated preferences. This may reflect how individuals process information, particularly in emotionally salient scenarios.

#### Hyperbolic discounting

The behavioural economic concept of hyperbolic discounting can be a powerful driver of sub-optimal decision-making. This bias describes the dynamics behind impatient behaviour, arguing that people are influenced more by present costs rather than distant benefits [84]. In life-limiting illness contexts, this can explain the lack of engagement in end-of-life planning, where the benefits of such conversations can be overridden by impact of immediate negative emotions such as decisional regret, anticipatory grief, mistrust, uncertainty, and fear of death [42, 62, 77].

For example, some oncologists prescribe chemotherapy at end of life to avoid difficult conversations and manage their discomfort with uncertainty [48]. This behaviour could be motivated by hyperbolic discounting, as the emotional cost of having a difficult conversation, which may diminish hope in the patient or threaten the identity of the oncologist, is burdensome and immediate [47]. Meanwhile, the benefits of such conversations, including having your wishes honoured and reducing family carer burden, are abstract and far in the future. Similar behaviour has been shown amongst nephrologists, who report agreement with shared-decision making yet express difficulty in discussing palliative care [65]. Interventions such as advance care planning may manage hyperbolic discounting by encouraging conversations about end of life. This may reduce undesired aggressive end-of-life care and ease carer burden by minimising uncertainty [57, 70].

However, hyperbolic discounting can provide insights into why individuals may avoid advance care planning. This is supported by Huffman et al., (2019) [29], who found that cognitive impairment is associated with impatience, which is associated with less end-of-life planning. Hyperbolic discounting can be further identified in study by Van de Water et al., (2024) [80], where individuals reported negative emotions in the week following shared decision-making. They argued this arose from the loss of hope and need to re-anchor expectations around life expectancy. However, this effect was temporary, indicating that the costs generated by such conversations only have a short-term impact.

#### Empathy gap

The empathy gap describes the phenomena of individuals failing to appreciate how their current environment affects their preferences and decisions [85]. The interpersonal gap arises when individuals inaccurately predict their preferences regarding a scenario they are not directly experiencing.

Surrogate decision-makers may project their current preferences onto hypothetical future scenarios that do not align with the patient’s preferences [35, 60]. Surrogates tend to systematically underestimate health related quality-of-life (HRQoL) in comparison to patients’ self-reported HRQoL, which complicates their ability to make informed decisions about care [86]. This represents a significant measurement issue, highlighting how poorly positioned surrogate decision-makers are to make accurate care decisions when they are distanced from the patient’s experience. Dening et al., (2016) [60] found greater concordance between people with dementia and their informal carers when discussing present health status scenarios, compared to hypothetical future scenarios where there was less uncertainty about predicting preferences. This reflects not only the empathy gap but also the broader challenge of predicting future preferences under uncertainty, especially in degenerative conditions like dementia.

#### Non-linear value of time

There is a well-documented paradox in the economics literature of futile high spending on end-of-life care that offers minimal life extension [27]. Economics researchers have attempted to rationalise this spending by arguing the value of a life year increases in proximity to death [23, 36, 40].

Becker et al., (2007) [23] and Philipson et al., (2010) [36] argue the importance of hope at end of life drives high expenditures. They define hope as the ‘current consumption of future survival’, proposing that even marginal gains in time allow patients to hope to live long enough for a cure to be discovered [23].

However, this definition of hope is rooted in diseases like cancer and HIV, where life extension or cures are more plausible. In dementia, where decline is irreversible, hope is defined more by the quality, rather than the quantity, of life. Patients and informal carers may hope for preference-concordant care, pain management, emotional and religious support, and the opportunity for reconciliation or finding peace [49, 53, 56, 73, 81]. Thus, incorporating this clinical context into Becker et al.’s (2007) [23] theory, the seemingly futile spending on end-of-life care that does not prolong life is justified by the increasing hope for holistic dementia care.

## Discussion

### Main results

This narrative review of studies on decision-making with life-limiting illness identified six key economic themes that are relevant for dementia: (a) principal-agent problem [26], (b) uncertainty [51], (c) asymmetric information [13], (d) hyperbolic discounting [84], (e) the empathy gap [85], and (f) non-linear value of time [23]. These economic concepts were identified from the economics literature and applied as interpretive lenses across the broader evidence base, with reflections from PPI members used to refine interpretations for relevance to decision-making in dementia care. Through this narrative synthesis, we provide structured summaries of the literature while offering insight into understanding extant phenomena in the evidence base including high end-of-life costs and variable efficacy of advance care planning.

These results have implications for future economics research. Just 16% of the included studies published in economics journals addressed dementia, compared to 51% of studies published in health and social care journals. Most studies published in economics journals were US-based (56% vs. 32% in health and social care journals), while only 4% were UK-based, compared to 27% of health and social care studies. More economics research is needed to better understand decision-making in dementia care in the UK. Key economic concepts such as the principal-agent problem, uncertainty, asymmetric information, hyperbolic discounting, the empathy gap, and non-linear value of time were used to reveal decision-making dynamics.

### Modifiability of costs of dementia care

We collated and synthesised the literature to explore how an economic mode of thinking can improve understanding of decision-making in dementia care. At a time of growing costs and demand for dementia care, this evidence can provide insight into the microeconomic behaviours that drive formal, social, and household costs.

The findings of this review suggest that formal care costs, such as costs of time spent in hospital and life-extending medications, could be reduced in dementia care, saving resources without compromising patient outcomes for pareto optimal improvements. The paradox of high expenditures on care with limited survival benefit has attracted substantial attention [27]. Historically, economists have tried to rationalise such spending, pointing to the increased value of hope at end of life, but it seems more likely to be the result of sub-optimal decision-making [23, 36, 87]. We find evidence of information asymmetry and unrealised preferences, which lead to fragmented care and poor outcomes. Equipped with this new economic perspective on the dynamics that drive spending, we can develop interventions to modify such costs. For example, this review finds that health and social care practitioners respond particularly poorly to uncertainty and emotionally burdensome conversations, which can lead them to overprescribe aggressive and costly end-of-life treatments. Medical education could be adapted to support individuals with managing the uncertainties of end-of-life care, which may promote the increased use of advance care planning, consequently reducing expenditures on costly and undesired treatments. Patient safety interventions, which can include training health and social care practitioners, are an additional means to reduce overprescribing. Such interventions could be particularly relevant in dementia, where the overprescribing of antipsychotic medications is both costly and associated with adverse outcomes such as pneumonia, acute kidney injury, and stroke [88, 89].

The modifiability of social costs is less clear. Interventions aimed at delaying entry into long-term care facilities have had limited success [90]. This may be due to the potential inevitable demand for such facilities but could partly reflect the methodological challenge of evaluating non-pharmacological interventions for people with dementia. Outcome measurements in such evaluations are often inconsistent with the preferences of people living with dementia, who are rarely consulted in identifying what outcomes should be prioritised [91]. In this context, there is limited evidence of long-term benefits and reliance on proxy-reported health utility [92]. Recent evidence on combined utility values, calculated by integrating self- and proxy-reported EQ-5D-5L, has been shown to be more accurate for populations with mild-to-moderate dementia, but evidence in severe dementia remains lacking [93]. This is consistent with recognised limitations of QALYs in palliative care, arising from the assumption that time has a constant and additive value at the end of life [94]. Earlier dementia diagnosis may reduce social costs, yet dementia remains underreported due to diagnostic uncertainty, limited incentives for health and social care practitioners, and limited family carer literacy [95]. Regardless, as dementia progresses, individuals inevitably reach a stage where they require constant support, with social care costs associated with severe dementia being triple those of individuals with mild dementia [6]. Furthermore, data on social care costs is inconsistently measured, which further complicates our ability to understand opportunities for cost-saving within this sector [6].

Similarly, the household costs of informal care are less obviously modifiable. Household costs refer to costs associated with informal care, as well as out-of-pocket spending, productivity losses, hours spent providing care and diminishing assets [3]. Evidence shows that there is to some extent a substitution effect between formal or social, and informal care, suggesting that greater provision of formal and social care can reduce the supply of informal care, with the potential to increase productivity [96, 97]. However, this does not necessarily reduce overall expenditures but often merely shifts where costs are imposed. The costs of informal care are particularly challenging to estimate, due to the complexity of the opportunity costs involved and the risk of overreporting. The replacement cost method values an hour of informal care using a market substitute, such as home care, treating informal care as providing a job that would otherwise be provided by a healthcare service [98]. Alternatively, informal care can be valued as the opportunity cost, incorporating foregone work and leisure [99]. However, the importance of supportive and holistic dementia care identified in this review suggest that spending time with a loved one and being available for support is valued by people living and dying with dementia. Where preferences for time with a loved one are lexicographical, an opportunity cost for this activity cannot be calculated. Population ageing means that demand for family carers will increase, yet the supply will decrease [100]. Therefore, future economics research should identify the extent to which we can modify social and household costs, which account for 84% of the global societal costs of dementia [3].

While a large proportion of such costs are less obviously modifiable, this review demonstrates that economic insights remain highly relevant to understanding how such costs arise. Many high-cost outcomes reflect decision-making under uncertainty and misaligned preferences rather than unavoidable clinical necessity. The impact of this work therefore lies not in identifying straightforward cost reductions, but in clarifying where improved communication, preference elicitation, and support for decision-makers may reduce non-beneficial care and improve quality of life for people living with dementia.

### Further research opportunities

This review identified the complexities of asymmetric information in dementia and life-limiting illness contexts, which if addressed could mitigate the costs of dementia. We found asymmetric information between patients, health and social care practitioners, and informal carers, but this can also persist amongst health and social care practitioners. This can be evidenced during transitions of care. Medication errors, which often arise from moving between care settings, are estimated to cost the NHS £98 million per year [101]. This may especially impact people with dementia, who often move between multiple care settings like the home, care home, hospice, and hospital [102]. Communication between health and social care practitioners, potentially supported by a transition to a single digital record across settings, could reduce this modifiable cost.

In addition, asymmetric information can reveal the dynamics underscoring advance care planning. The primary purpose of advance care planning is to manage information asymmetry by sharing patient preferences and values with family carers and health and social care practitioners. By encouraging dynamic communication, this can mitigate the issues that arise with the principal-agent problem. Further, this intervention could address the uncertainties that are burdensome to decision-makers, potentially improving carer quality-of-life.

However, we also find negative evidence for advance care planning, including that it is not referred to, or carers continue to override patient preferences with their own [35, 53, 72]. This finding has been replicated in the wider literature [103, 104]. This may reflect the empathy gap, as surrogate decision-makers do not directly experience the health state of the person living and dying with dementia, which may generate differences in their preferences. Evidence suggests that surrogate decision-makers tend to overestimate preferences for life-extending treatment while underestimating patients’ quality of life [86, 105, 106]. In the absence of documented preferences, surrogates must make decisions under considerable uncertainty, leading to fear of undertreatment. Further, studies exploring such differences often ask participants for preferences on hypothetical scenarios, while the participants are often members of the public rather than family carers [35, 60, 74]. This review also identified hyperbolic discounting as a deterrent to end-of-life planning.

If the dynamics which hinder advance care planning are further explored with decision-makers experienced in caring for people living and dying with dementia, economics could have the potential to provide a new perspective on advance care planning and explore the drivers of costs. Future research could explore means to manage these biases by examining the presence of the empathy gap in advance care planning, for example by comparing outcomes for individuals whose advance care planning records were developed with and without the involvement of family carers. This could promote advance care planning as an intervention which is used to achieve preference-concordant care, minimise decisional regret for informal carers, and reduce end-of-life spending.

We identified literature which attempts to rationalise what is often critiqued as futile spending on end-of-life care by arguing the value of hope increases towards end of life. Similarly, the red herring hypothesis argues that proximity to death, not age, drives end-of-life costs [107, 108]. This suggests that high spending is not irrational but rather reflects intensified needs as death approaches. In dementia, needs often include non-pharmacological support which are underrepresented in CEA.

Framing high end-of-life spending as a reflection of underlying values and perceptions, rather than as a waste of resources, suggests that this is a proxy for decision-making under uncertainty, asymmetric information, and emotional salience. In this light, understanding spending can reveal the underlying priorities that shape how decisions are made in life-limiting illness, while also highlighting why spending patterns may look different in the context of dementia.

### Strengths and limitations

A strength of this study was the involvement of PPI. People living with dementia and family carers were invited to discuss the preliminary findings of this study. For example, they highlighted that decision-making models are oversimplified, instead emphasising the complexity and number of decision-makers involved in dementia care. They also argued the models have varying applicability depending on the stage of dementia, suggesting that modelling autonomous patient-led decisions are likely to be more representative at an earlier stage of dementia. These contributions offered nuance beyond empirical evidence, and we will continue to engage with PPI members in the dissemination of this work and development of future research. The systematic search found no economics studies incorporating PPI, compared to 14% of health and social care papers. This is consistent with the wider literature, which find the movement towards PPI involvement in health and social care research is not being replicated with the same momentum in economics research [109]. It is important to tap into this rich source of experience to ensure real-world relevance. Given that we identify the modifiability of social and household costs, which are often determined by individual decisions regarding the location and amount of care received, as a key direction for future research, it could be argued that incorporating PPI is not only beneficial but essential.

The main limitation of this study is that it is not known whether insights from behavioural economics, a discipline exploring cognitive biases, can be applied to the behaviour of people with cognitive decline. This study has proposed economic concepts which seem relevant to the reality evidenced in the health and social care literature and PPI discussions, yet future research should empirically test these biases within dementia populations. The PPI members involved in this study were people living with dementia and family carers and therefore did not capture the perspective of health and social care practitioners. Future research would benefit from broader PPI involvement spanning key stakeholder groups across the dementia care pathway, including clinicians, care providers, and policymakers. Our search strategy has limitations, as title/abstract screening was conducted by one reviewer, with a second reviewer being introduced for full-text screening. In addition, we categorised studies as economics or health and social care based on journal discipline to explore patterns of research across the two fields. However, we acknowledge that economics research may also be published in health and social care journals. The included studies were conducted across countries with differing health economic policies and incentive structures, which should be considered when interpreting the findings. Finally, to apply economic concepts across heterogeneous diseases, we broadened the scope of our search to include life-limiting illness, but it is possible that we could have gained valuable insights from considering more widely older people with frailty who may be approaching the end of life and populations such as care home residents. Future research could build on our findings in this broader context.

## Conclusion

This study compiles and synthesises the literature to apply an economic mode of thinking to decision-making in dementia care. It seeks to advance how decisions in this setting are understood and made by examining how economic concepts can inform interactions between the multiple actors involved in dementia care. By integrating economic theory with clinical evidence, the study explores the applicability of economic concepts exploring decision-making to patients who are cognitively constrained. We identify concepts such as the principal-agent problem, uncertainty, asymmetric information, hyperbolic discounting, the empathy gap, and non-linear value of time. We use these concepts to understand the biases within individual decision-making that may explain the negative results for advance care planning and recognise drivers of high end-of-life expenditures. Using these insights, we propose that economic insights are more obviously applicable to reducing formal healthcare costs, but it is less clear whether they can have a big effect on social and household costs, which is where the majority of dementia costs lie. While many dementia-related costs are less obviously modifiable, this review demonstrates how economic insights can clarify the mechanisms through which such costs arise. Ultimately, this research proposes that developing our understanding of how patients, health and social care practitioners, and family carers make decisions can inform strategies to deliver preference-concordant care and minimise the costs of dementia care.

## Supplementary Information

Supplementary Material 1.

## Data Availability

No datasets were generated or analysed during the current study.
