# Supplementary material for: The economics of decision-making in dementia care and related life-limiting illness: a narrative review
Source: Health Econ Rev. 2026 Jun 5;16:96. doi: 10.1186/s13561-026-00803-2 (PMC13465789; doi:10.1186/s13561-026-00803-2)
Supplement: Supplementary file 1 [file 13561_2026_803_MOESM1_ESM.docx]

**Supplementary Materials**

**Systematic search categories**

Table 2 Systematic search categories

| Database(s) | Search category 1 | Boolean Operator | Search category 2 | Boolean Operator | Search category 3 |
| --- | --- | --- | --- | --- | --- |
| MedLine, Embase, PsycInfo | Life-Limiting Illnesses | AND | Type of Illness | AND | Shared Decision-Making |
| EconLit | Life-Limiting Illnesses | OR | Type of Illness | AND | Decision-Making |

**Systematic search: Search terms**

*MedLine, Embase, PsychInfo via OVID*

tw = title/abstract

1. ("palliative" or "hospice" or "end of life" or "serious illness*" or "terminal illness*" or "terminal care" or "family care*" or "informal care*" or "life threatening illness*" or "serious chronic illness*" or "serious medical illness*" or "life limiting illness*" or "life limiting disease" or "advanced illness" or "life threatening disease*" or "serious disease*" or "serious chronic disease*" or "serious medical disease*" or "advanced disease*").tw.
2. limit 1 to yr="1860 - 2024"
3. ("multimorbid*" or "comorbid*" or "cognitive impairment" or "cognitively impaired" or "cognitive decline" or "cancer" or "dementia" or "renal" or "liver" or "alzheimer's disease" or "ADRD" or "serious heart disease" or "kidney*" or "cirrhosis" or "COPD" or "breathlessness" or "AIDS" or "HIV" or "chronic obstructive pulmonary" or "huntington's disease" or "motor neurone disease" or "parkinson's disease" or "multiple sclerosis").tw.
4. limit 3 to yr="1860 - 2024"
5. ("shared decision making" or "proxy decision making" or "surrogate decision making" or "dyad* decision making" or "triad* decision making" or "decision rules" or "decisional capacity" or "treatment decision*" or "decision science" or "principal agen*" or "agency problem" or "microeconomic*" or "rational*" or "value of time" or "value of hope" or "asymmetric information" or "moral hazard" or "incentive" or "positive theory").tw.
6. limit 5 to yr="1860 - 2024"
7. 2 and 4 and 6

*EconLit via ProQuest*

NOFT = anywhere except full text

1. ("palliative" OR "hospice" OR "end of life" OR "serious illness*" OR "terminal illness*" OR "terminal care" OR "family care*" OR "informal care*" OR "life threatening illness*" OR " serious chronic illness*" OR "serious medical illness*" OR  "life limiting illness*" OR "life limiting disease*" OR "advanced illness" OR "life threatening disease*" OR "serious disease*" OR "serious chronic disease*" OR "serious medical disease*" OR "advanced disease*" OR "multimorbid*" OR "comorbid*" OR "cognitive impairment" OR "cognitively impaired" OR "cognitive decline" OR "cancer" OR "dementia" OR "renal" OR "liver" OR "alzheimer’s disease" OR "ADRD" OR "serious heart disease" OR "kidney*" OR "cirrhosis" OR "COPD" OR "breathlessness" OR "AIDS" OR "HIV" OR "chronic obstructive pulmonary" OR "huntington’s disease" OR "motor neurone disease" OR "parkinson’s disease" OR "multiple sclerosis") NOFT
2. limit 1 to yr="1964 - 2024"
3. ("decision making" OR "decision maker" OR "decision rules" OR "decisional capacity" OR "treatment decision*" OR "decision science" OR "principal agen*" OR "agency problem" OR "microeconomic*" OR "rational*" OR "value of time" OR "value of hope" OR "asymmetric information" OR "moral hazard" OR "incentive" OR "positive theory") NOFT
4. limit 3 to yr="1889 - 2024"
5. 1 and 2
